# Emerging and Novel Ovine Viruses: Molecular Diagnostics, Genomic and Metagenomic Surveillance, and One Health Perspectives

**DOI:** 10.3390/ani16193066

**Published:** 2026-09-29

**Authors:** Gul Muhammad Shahbaz, Zeeshan Ashraf, Shuxin Chen, Chaofan Wang, Chenglong He, Huiping Sun, Lexiao Zhu, Lei Liu, Mingcheng Wang, Linglong Wu, Ruohuai Gu, Wei Li, Feng Xing

**Affiliations:** 1 Key Laboratory of Tarim Animal Husbandry Science and Technology, Xinjiang Production and Construction Group, School of Animal Science and Technology, Tarim University, Alaer 843300, China; shahbazbalouch42@gmail.com (G.M.S.); 13707528852@163.com (S.C.); 13333986752@163.com (C.W.); h42320213@163.com (C.H.); sunhuiping223@163.com (H.S.); 18538512047@163.com (L.Z.); 13547040655@163.com (L.L.); wmcanjiaowangdidan@163.com (M.W.); 18597814328@163.com (W.L.); 2 Key Laboratory of Livestock and Forage Resources Utilization around Tarim, Ministry of Agriculture and Rural Affairs, School of Animal Science and Technology, Tarim University, Alar 843300, China; zeeshanashraf1062@gmail.com

**Keywords:** Ovine viruses, emerging viral diseases, molecular diagnostics, genomic surveillance, viral metagenomics, nanopore sequencing, One Health, veterinary epidemiology

## Abstract

Emerging and novel ovine viruses pose increasing threats to animal health, livestock productivity, trade, food security, and public health preparedness. Their emergence is driven by complex interactions among animal movement, mixed-species production systems, wildlife–livestock interfaces, arthropod vectors, environmental changes, and viral evolution. This review critically compares current strategies for identifying major, emerging, re-emerging, zoonotic, and newly recognized ovine viruses, with emphasis on their analytical sensitivity, turnaround time, throughput, operational cost, accessibility, field applicability, validation status, and capacity for novel-virus detection. Conventional diagnostic approaches, including virus isolation, serology, antigen detection, histopathology, and immunohistochemistry, remain essential for confirmation and flock-level surveillance but may be limited by slow turnaround, dependence on specialized facilities, reduced sensitivity at low viral loads, and an inability to identify highly divergent or unknown viruses. Targeted molecular assays, including PCR, RT-PCR, qPCR, multiplex assays, digital PCR, isothermal amplification, and CRISPR-based diagnostics, have improved detection speed and sensitivity but generally require prior knowledge of viral genomic targets. Genomic and metagenomic approaches, including whole-genome sequencing, next-generation sequencing, nanopore sequencing, viral metagenomics, bioinformatics, and phylogenetic analysis, provide broader detection capabilities by enabling characterization of viral diversity, outbreak tracing, co-infection identification, and discovery of previously unrecognized viruses. However, their interpretation remains challenging due to low viral abundance, poor sample quality, host nucleic acid background, contamination, incomplete reference databases, limited computational capacity, and the inability of sequence detection alone to confirm disease causality. Therefore, future ovine virus surveillance requires integration of molecular diagnostics with active, passive, outbreak-based, risk-based, vector, wildlife, and animal-movement surveillance within a One Health framework. Linking genomic information with ecological, epidemiological, and environmental data will be essential for transforming ovine virus surveillance from reactive diagnosis toward proactive preparedness. Advances in standardized sampling, validated field diagnostics, affordable sequencing, curated databases, bioinformatics capacity, and cross-sector data sharing will strengthen early recognition, risk assessment, and preparedness against emerging viral threats in sheep.

## Simple Summary

Emerging and novel viral infections in sheep represent important challenges for animal health, sustainable livestock production, and disease preparedness. The increasing movement of animals, wildlife–livestock interactions, changes in vector distribution, and environmental pressures create opportunities for the emergence and spread of new viral threats. Although conventional diagnostic methods remain crucial for identifying known infections, they may be insufficient for detecting highly divergent or previously unrecognized viruses. This review discusses how molecular diagnostics, genomic surveillance, and metagenomic approaches are transforming the identification and monitoring of ovine viruses by enabling broader virus detection, genetic characterization, and investigation of transmission patterns. However, effective use of these technologies requires accurate sampling, reliable data interpretation, and integration with veterinary epidemiology and One Health surveillance. Combining genomic information with animal health monitoring, wildlife surveillance, and environmental data will improve early recognition of emerging viral threats and strengthen strategies for protecting sheep production systems and animal health.

## 1. Introduction

Sheep are raised across diverse production systems and contribute to meat, milk, and wool production worldwide [1]. In many regions, sheep are maintained in close contact with other livestock species, wildlife, humans, arthropod vectors, and shared environments, creating opportunities for pathogen maintenance, evolution, and transmission [2]. Because viral emergence increasingly occurs at the animal–human–environment interface, early recognition of emerging and novel ovine viruses has become essential for protecting animal health, ensuring food security, improving disease control, and strengthening public-health preparedness [3]. Several ovine viruses have considerable veterinary, economic, and epidemiological importance, including sheep pox virus [4], orf virus [5], peste des petits ruminants virus (PPRV) [6], bluetongue virus (BTV) [7], border disease virus [8], maedi-visna virus [9], Rift Valley fever virus (RVFV) [10], and jaagsiekte sheep retrovirus [11]. These viruses are associated with diverse clinical outcomes, including skin lesions, respiratory disease, reproductive failure, congenital disorders, immunosuppression, production losses, mortality, and trade restrictions. Together, these infections illustrate the diverse mechanisms underlying ovine viral emergence, including transboundary spread, vector expansion, persistent infection, zoonotic spillover, and cross-species transmission. Increasing animal movement, climate change, wildlife–livestock interactions, mixed farming systems, and viral genetic evolution further increase the possibility of new variants and unexpected viral events in sheep populations [7]. Traditional diagnostic approaches, including virus isolation, serology, antigen detection, histopathology, and electron microscopy, remain important for confirming ovine viral infections. However, these methods may require specialized facilities, have limited turnaround times, or fail to identify highly divergent and previously unknown viruses. Molecular techniques, including PCR, RT-PCR, quantitative real-time PCR, multiplex PCR, digital PCR, loop-mediated isothermal amplification, and CRISPR-based assays, have substantially improved the speed, sensitivity, and specificity of viral detection in veterinary medicine [12]. Nevertheless, most targeted molecular assays rely on prior knowledge of viral genomic sequences, creating a major limitation during unexplained outbreaks where the causative virus is unknown or genetically divergent [13]. Genomic and metagenomic technologies have therefore transformed virus discovery and surveillance by extending detection beyond predefined diagnostic targets. Whole-genome sequencing supports strain characterization, mutation analysis, phylogenetic investigation, molecular epidemiology, and outbreak tracing, whereas viral metagenomics enables sequence-independent detection of known, divergent, and previously unrecognized viruses [14]. Recent studies have demonstrated the value of next-generation sequencing and metagenomic approaches for identifying unexpected viruses in sheep, including respiratory-associated viruses, co-infections, and novel ovine viral sequences [15]. Importantly, these approaches provide an opportunity to move ovine virus surveillance from reactive detection of known pathogens toward proactive recognition of emerging viral threats. However, sequence-based discoveries require careful interpretation through integration with clinical findings, epidemiological information, and biological confirmation. A One Health perspective is essential for understanding emerging and novel ovine viruses because sheep are embedded within interconnected livestock, wildlife, vector, human, and environmental systems [3]. Ovine-associated viruses such as RVFV and orf virus have direct zoonotic importance, whereas other viruses may influence public health indirectly through livestock productivity, food security, and rural economies [10]. Therefore, effective surveillance requires integration of veterinary diagnostics, public-health systems, wildlife monitoring, genomic databases, ecological information, and epidemiological networks rather than relying on livestock-based detection alone [2]. This review critically evaluates the identification and detection of emerging and novel ovine viruses, focusing on molecular diagnostics, genomic and metagenomic surveillance, bioinformatics analysis, veterinary epidemiology, and One Health integration. Specifically, this review examines current diagnostic capabilities, the opportunities and limitations of sequence-based virus discovery, the role of integrated surveillance frameworks, and future strategies for improving early recognition and preparedness against emerging viral threats in sheep.

## 2. Methods

This narrative review drew on the literature identified through PubMed/MEDLINE, Google Scholar, and the NIH Bookshelf. Searches addressed viruses affecting sheep, including established, emerging, and newly detected agents, together with molecular diagnosis, genomic and metagenomic sequencing, epidemiology, and One Health surveillance. The searches returned 69 records in PubMed/MEDLINE, approximately 18,300 results in Google Scholar, and 63 records in the NIH Bookshelf. These figures represent results displayed by each source; the Google Scholar count is approximate, and results from different sources may overlap. Relevant publications and their reference lists were examined to identify evidence addressing the review’s principal themes.

Publications were selected for their relevance to ovine viral disease, diagnostic methods, genomic characterization, unexpected virus detection, or surveillance involving animals, humans, vectors, and the environment. Original studies and outbreak investigations were prioritized when discussing specific viruses, assay performance, and sequencing findings. Reviews and authoritative guidance provided background and broader context. Evidence from other animal species was included when directly relevant to diagnostic approaches or potential cross-species exposure, while its applicability to sheep was considered separately. In evaluating newly detected viruses, identification of viral nucleic acid was distinguished from evidence of active infection, disease causation, or transmission between species. The literature was synthesized narratively across four themes: ovine viral emergence, conventional and molecular diagnosis, genomic and metagenomic detection, and integrated surveillance. Studies differed substantially in their pathogens, specimens, methods, and outcomes; therefore, no quantitative meta-analysis was performed. The final review cites 83 references used to support its discussion and interpretation.

## 3. Emerging and Novel Viruses in Sheep

Sheep are susceptible to a broad range of viral pathogens, including endemic, emerging, re-emerging, vector-borne, zoonotic, and newly detected viruses. These viruses are crucial not only because they cause clinical disease, but also because they affect livestock productivity, animal trade, outbreak preparedness, and One Health surveillance. When considering novel virus detection, sheep represent an important host species for studying viral emergence, cross-species transmission, genetic diversity, and the application of molecular and metagenomic diagnostic tools. However, ovine viral emergence is driven by complex interactions among viral evolution, livestock production systems, animal movement, wildlife contact, vector ecology, and environmental changes. Although advances in molecular diagnostics and sequencing technologies have improved detection capacity, many surveillance systems remain primarily focused on known pathogens, creating challenges for early recognition of divergent or previously unrecognized viruses [7,15]. Therefore, understanding the ecological and epidemiological drivers of viral emergence is essential for developing proactive surveillance strategies in sheep populations. Sheep pox virus is one of the most important viral pathogens in sheep and belongs to the genus *Capripoxvirus*. It is a contagious disease characterized by fever, skin lesions, respiratory signs, lymph node enlargement, and variable mortality, particularly in susceptible populations. The disease is highly relevant for veterinary surveillance because of its economic impact, transboundary potential, and requirement for rapid laboratory confirmation during outbreaks. Molecular detection and genomic characterization assist in confirming infection, differentiating related capripoxviruses, and supporting epidemiological tracing. However, the transboundary nature of capripoxvirus infections highlights a major limitation of outbreak-based surveillance, where detection may occur only after substantial viral circulation has already occurred. Genomic approaches improve outbreak tracing and source attribution, but their effectiveness depends on timely sampling, sequencing capacity, and integration with epidemiological information [16]. Orf virus, a member of the genus *Parapoxvirus*, causes contagious ecthyma in sheep and goats and has clear One Health importance because it can infect humans through direct contact with infected animals or contaminated materials. Although infection is often self-limiting, outbreaks may result in economic losses associated with reduced feeding, secondary bacterial infections, poor growth, and lamb mortality. Because clinical lesions may resemble other vesicular or proliferative skin diseases, laboratory confirmation is essential. Current diagnostic approaches include PCR, real-time PCR, LAMP, recombinase-based amplification, serological testing, and sequencing-based methods [17]. Peste des petits ruminants virus is a highly contagious morbillivirus affecting sheep and goats and remains a major threat to small-ruminant production. The disease is associated with fever, ocular and nasal discharge, oral lesions, diarrhea, pneumonia, high morbidity, and variable mortality. Its transboundary nature makes rapid detection and genomic surveillance essential for outbreak investigation and control. Recent genomic analyses of PPR virus outbreaks demonstrated the value of sequencing for identifying outbreak origins and supporting molecular epidemiology. Nevertheless, genomic tracing is mainly a retrospective approach and has limited predictive capacity when not combined with continuous surveillance, animal movement information, and risk-based sampling. Therefore, genomic data should be integrated with epidemiological and ecological information to improve early-warning capacity rather than being used only after outbreaks occur [18]. Bluetongue virus is an important vector-borne pathogen transmitted mainly by *Culicoides* biting midges and affects both domestic and wild ruminants. Sheep are among the most clinically affected species, showing fever, facial edema, oral lesions, lameness, respiratory signs, and production losses. Bluetongue is particularly relevant to emerging virus surveillance because its distribution is influenced by vector ecology, climate conditions, animal movement, novel serotypes, and viral reassortment. Molecular detection, serotyping, whole-genome sequencing, and vector surveillance are therefore important for monitoring emergence in sheep populations [19].

Bluetongue virus represents a model example of climate-sensitive viral emergence, demonstrating that effective surveillance requires integration of pathogen genomic data with vector monitoring, environmental information, and livestock movement patterns rather than relying solely on clinical diagnosis. Border disease virus is a pestivirus of sheep that may cause reproductive failure, abortion, stillbirth, weak lambs, congenital abnormalities, and persistent infection. Persistently infected sheep are epidemiologically important because they can maintain and transmit infection within flocks. Molecular surveillance in mobile flocks and traditional grazing systems has demonstrated the importance of genetic characterization for understanding virus circulation. PCR, serology, virus neutralization, and phylogenetic analysis are useful for confirming infection and transmission patterns [20]. Maedi-visna virus, a small ruminant lentivirus, causes chronic progressive disease mainly affecting the lungs, mammary gland, joints, and central nervous system. The long incubation period, persistent infection, and slow clinical progression make detection and control difficult. Serological screening remains important for flock-level surveillance, whereas PCR-based approaches assist confirmation and tissue-level detection. Because infections may remain subclinical for prolonged periods, repeated surveillance and improved diagnostic strategies are required for effective control [21]. Together, chronic and persistent infections such as border disease virus and small ruminant lentiviruses demonstrate that surveillance challenges are not limited to newly introduced viruses. Long-term circulation of genetically diverse viruses within apparently healthy populations represents an additional challenge requiring continuous monitoring and complementary diagnostic approaches [20,21]. Rift Valley fever virus is a mosquito-borne zoonotic virus affecting sheep, goats, cattle, wildlife, and humans. In sheep, infection can cause abortion storms, neonatal mortality, fever, and high mortality in young animals. Its zoonotic potential makes it highly relevant to One Health surveillance. Recent outbreaks have emphasized the importance of coordinated responses involving molecular diagnosis, serology, vector monitoring, animal movement control, and risk-based surveillance. The epidemiology of Rift Valley fever demonstrates that ovine viral emergence cannot be considered solely a livestock health problem because environmental conditions, vector dynamics, and human exposure pathways influence outbreak risk and transmission potential [22]. Jaagsiekte sheep retrovirus is the causative agent of ovine pulmonary adenocarcinoma, a contagious lung tumor of sheep. Affected animals may show chronic respiratory signs, progressive weight loss, and reduced productivity, whereas early infection can be difficult to detect. Diagnosis may involve clinical examination, imaging, histopathology, immunohistochemistry, PCR-based detection, and post-mortem confirmation. From a surveillance perspective, jaagsiekte sheep retrovirus illustrates the diagnostic challenges associated with persistent and slowly progressive viral infections [23]. In addition to recognized ovine viruses, sequencing studies have demonstrated that sheep may harbor unexpected or previously unrecognized viruses. Next-generation sequencing identified bovine rhinitis B virus and a novel ovine nidovirus from respiratory samples of sheep, suggesting possible cross-species transmission or spillover in mixed farming systems [24]. Similarly, detection of influenza A(H5N1) of avian origin in sheep through routine surveillance highlights the importance of monitoring unexpected viral infections in livestock populations [25]. These findings demonstrate that sheep should be considered not only as hosts affected by viral diseases but also as potential sentinel animals for detecting unexpected viral circulation at livestock–wildlife and multispecies farming interfaces. However, interpretation of novel viral sequences remains challenging because detection alone does not confirm active infection or disease causality; therefore, genomic findings require integration with clinical, pathological, and epidemiological evidence [24,25]. Collectively, emerging, re-emerging, and newly detected ovine viruses demonstrate that viral emergence occurs through diverse mechanisms, including transboundary movement, climate-driven vector expansion, persistent infection, and cross-species spillover. Consequently, future surveillance strategies should move beyond virus-specific detection toward integrated frameworks combining clinical investigation, molecular diagnostics, genomic sequencing, metagenomics, bioinformatics, and epidemiological interpretation to achieve earlier recognition and improved preparedness for future viral threats. The principal clinical importance, diagnostic approaches, and surveillance and One Health relevance of major established, emerging, and newly detected ovine viruses are summarized in Table 1.

## 4. Conventional and Molecular Diagnostic Methods

Many ovine viral diseases share similar clinical manifestations, including fever, respiratory signs, skin lesions, reproductive disorders, diarrhea, neurological abnormalities, and reduced productivity. Therefore, clinical examination alone is insufficient for accurate identification, particularly during outbreaks or when an unfamiliar viral agent is suspected. Reliable detection of emerging and novel ovine viruses requires integration of clinical evaluation with conventional diagnostic methods, serology, antigen detection, and molecular approaches [26]. However, the increasing diversity of ovine viral threats creates a major diagnostic challenge because traditional surveillance systems are mainly designed for known pathogens and may fail to recognize genetically divergent or unexpected viruses. Therefore, modern diagnostic strategies must balance rapid detection of known viruses with approaches capable of identifying novel viral agents. Virus isolation remains an important diagnostic method because it confirms the presence of infectious virus and supports virus characterization, antigenic studies, and vaccine development. It has been applied to important ovine viruses, including sheep pox virus, orf virus, peste des petits ruminants virus, and bluetongue virus. However, virus isolation is limited by long processing times, dependence on viable virus particles, sample quality, suitable cell culture systems, and biosafety requirements. Consequently, although virus isolation remains valuable in reference laboratories and research applications, it is increasingly used as a complementary method combined with molecular confirmation rather than as the primary approach for rapid outbreak diagnosis [27].

Serological assays are widely used for flock-level screening, exposure assessment, and retrospective diagnosis. Techniques such as enzyme-linked immunosorbent assays (ELISAs), virus-neutralization tests, agar gel immunodiffusion, complement fixation, and immunofluorescence assays have been applied for viruses including bluetongue virus, maedi-visna virus, border disease virus, and peste des petits ruminants virus [28]. Nevertheless, interpretation of serological results can be complicated by delayed antibody production, maternal antibodies, vaccination history, and cross-reactivity. Therefore, serological findings should be interpreted alongside clinical information, epidemiological data, and vaccination status rather than treated as direct evidence of active infection. Antigen detection methods, including antigen-capture ELISAs, lateral flow assays, and immunohistochemistry, provide rapid detection, particularly when viral antigen abundance is sufficient in clinical or post-mortem samples. These methods are advantageous for field applications because they are relatively simple and rapid. However, sensitivity may decrease when viral loads are low or antigens are degraded. Therefore, positive or uncertain results often require confirmation using molecular techniques, especially during outbreaks where accurate identification is essential for control measures [29]. PCR and reverse-transcription PCR (RT-PCR) have become central tools for direct detection of DNA and RNA viruses from various clinical samples, including blood, serum, swabs, milk, faeces, skin lesions, tissues, and aborted fetal materials. Amplicon sequencing can further support viral identification and genetic characterization, whereas conventional PCR remains useful for confirmation of suspected infections [30]. Real-time PCR and quantitative RT-PCR have further improved diagnostic capacity by enabling rapid detection and estimation of viral loads, making them valuable for outbreak response, quarantine decisions, epidemiological tracing, and monitoring of viral shedding in diseases such as peste des petits ruminants virus, sheep pox virus, bluetongue virus, and Rift Valley fever virus [31]. Despite their high sensitivity and specificity, targeted PCR-based assays have an important limitation: they require prior knowledge of viral genomic sequences. Therefore, they may fail to detect highly divergent, mutated, or completely novel viruses, creating a diagnostic gap during unexplained outbreaks. This limitation highlights the need for complementary approaches capable of detecting unknown viral agents. Although targeted molecular assays depend on prior sequence knowledge, this limitation can be partially mitigated through broader assay designs and enrichment strategies. Syndromic multiplex panels can include conserved targets from multiple viruses or virus families, reducing dependence on separate single-target assays [32]. Degenerate or consensus primers can accommodate sequence variation within conserved genomic regions and broaden detection of related strains [31]. Periodic comparison of primer and probe sequences with newly deposited genomes can also identify mismatches and guide assay updates. Tiling PCR uses overlapping amplicons across a viral genome and can support recovery and characterization of divergent or recombination-prone viruses when single-amplicon approaches are unsuccessful. Additional strategies include broad-range family-level PCR, sequence-independent amplification, random priming, viral enrichment, host nucleic acid depletion, and targeted enrichment followed by sequencing [14]. However, these approaches may introduce amplification or enrichment bias, reduce analytical sensitivity, require repeated primer redesign, or fail when target regions are highly divergent. Thus, they should be viewed as bridging strategies that broaden targeted detection rather than as replacements for untargeted metagenomic sequencing when the causative virus is genuinely unknown.

Among these mitigation strategies, multiplex PCR expands diagnostic capacity by allowing simultaneous detection of multiple predefined pathogens in a single reaction and is particularly useful when several viruses produce similar clinical syndromes. These assays reduce diagnostic time and sample requirements while supporting syndromic surveillance. However, multiplex platforms require careful optimization because target competition, assay complexity, and reduced sensitivity may affect performance [32]. Similarly, digital PCR provides absolute quantification of viral nucleic acids without standard curves and may be useful for low viral loads, weak-positive samples, persistent infections, and viral shedding studies. Nevertheless, high equipment costs, technical requirements, and limited standardization currently restrict routine application [33]. Isothermal amplification methods, such as loop-mediated isothermal amplification (LAMP), provide rapid detection without conventional thermocyclers and are suitable for field diagnosis, low-resource laboratories, and rapid outbreak responses. However, confirmatory testing may still be required for official surveillance purposes [34]. CRISPR-based diagnostic platforms represent an emerging approach with the potential for rapid and specific point-of-care detection. However, their performance is assay-dependent, and routine veterinary implementation requires virus-specific analytical validation, contamination-controlled workflows, standardized result interpretation, and field evaluation [35]. Collectively, these diagnostic platforms involve important trade-offs among analytical sensitivity, turnaround time, throughput, cost, accessibility, and field applicability. Conventional methods provide biological or serological evidence but may be slow or unsuitable for confirming early infection, whereas targeted molecular assays offer rapid and sensitive detection but remain dependent on predefined viral sequences. Genomic and metagenomic approaches provide broader characterization and greater capacity to investigate divergent or unexpected viruses, although their application requires greater sequencing, computational, and bioinformatic capacity. Therefore, platform selection should be guided by the surveillance objective, available infrastructure, sample characteristics, and whether the suspected viral agent is known or unanticipated. Overall, conventional and targeted molecular methods remain essential components of ovine virus diagnosis; however, no single platform is sufficient for identifying all emerging viral threats. The dependence of targeted assays on known sequences highlights the importance of integrating molecular diagnostics with genomic sequencing and metagenomic approaches. Therefore, a hierarchical diagnostic framework combining clinical investigation, conventional laboratory methods, molecular confirmation, and sequence-independent virus discovery represents a more comprehensive strategy for early recognition and surveillance of emerging and novel ovine viruses.

## 5. Genomic and Metagenomic Detection

**Figure 1:**
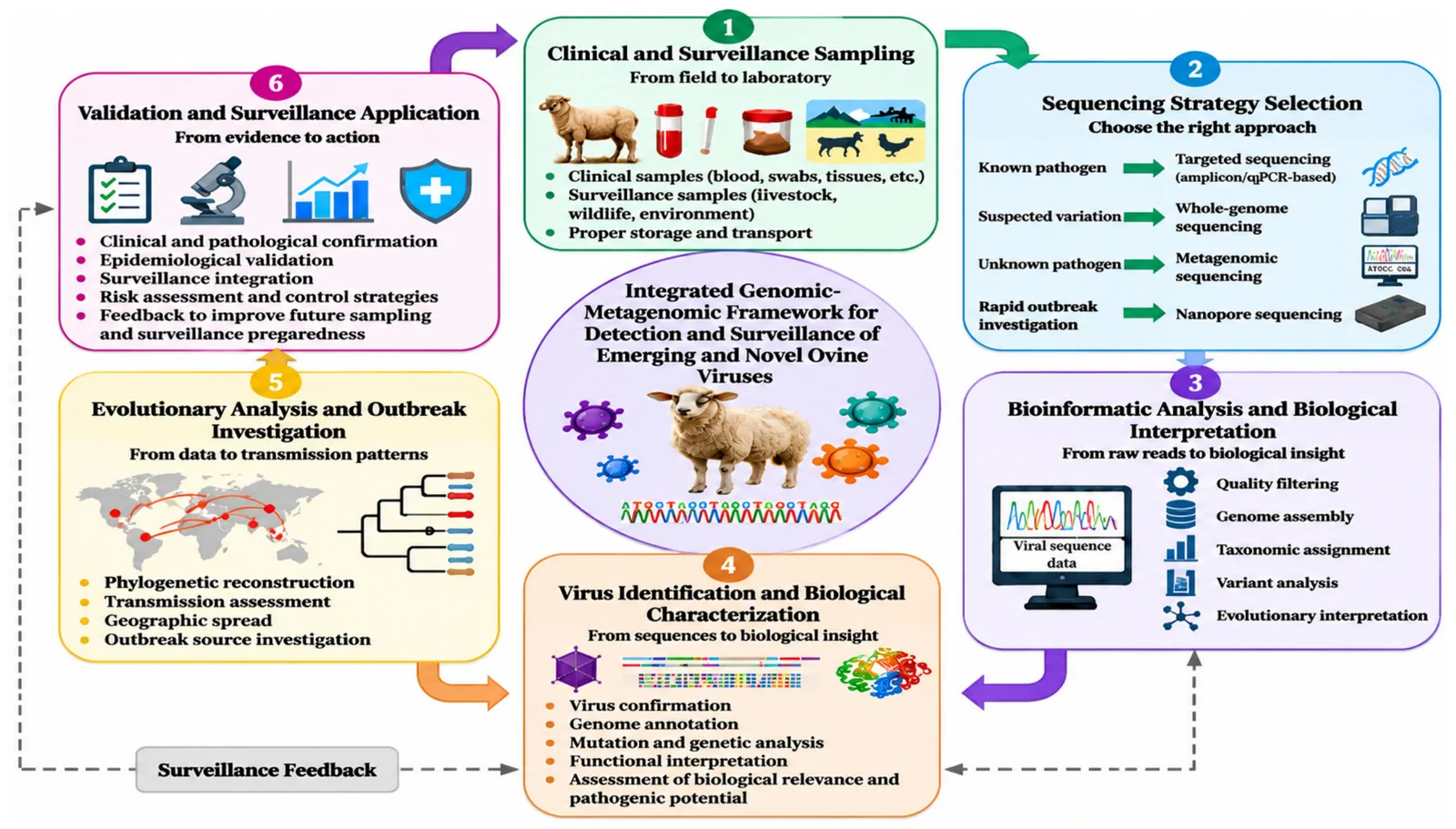
Integrated genomic–metagenomic framework for detection, characterization, and surveillance of emerging and novel ovine viruses. This framework illustrates the integration of clinical and surveillance sampling, sequencing approaches, bioinformatic analysis, viral identification, genomic characterization, phylogenetic interpretation, and epidemiological validation for emerging and novel ovine virus surveillance. The framework highlights the transition from targeted pathogen detection toward broader sequence-based virus discovery using whole-genome sequencing, next-generation sequencing, and metagenomic approaches. Solid arrows indicate the direction of analytical workflow and data interpretation, whereas dashed arrows represent feedback between surveillance findings and subsequent sampling or monitoring strategies. Different colors distinguish the major components of the framework, including sample acquisition, sequencing and bioinformatic analysis, viral characterization, and epidemiological integration.

Genomic and metagenomic technologies have become essential approaches for the detection and identification of emerging and novel ovine viruses. Unlike conventional targeted assays that depend on prior knowledge of viral sequences, sequencing-based approaches enable the detection of known, divergent, unexpected, and previously unrecognized viruses from clinical and surveillance samples. These approaches are particularly valuable during unexplained outbreaks, atypical clinical cases, mixed infections, and routine surveillance programs where the causative virus may not be anticipated. However, the major contribution of genomic and metagenomic technologies extends beyond improved detection sensitivity. Their ability to identify viruses without predefined genomic targets provides a pathway to overcome a major limitation of conventional diagnostics and supports a transition toward more proactive surveillance of emerging viral threats. Nevertheless, sequence-based findings require clinical and epidemiological interpretation to determine their biological significance [36]. The integrated workflow for sequence-based detection of emerging and novel ovine viruses, including sample collection, genomic and metagenomic sequencing, bioinformatic processing, virus identification, characterization, phylogenetic analysis, and epidemiological interpretation, is summarized in Figure 1.

Whole-genome sequencing (WGS) provides complete or near-complete viral genomic information and supports strain characterization, mutation analysis, molecular epidemiology, and outbreak tracing. During a 2024 sheep pox outbreak in Targap village, Almaty Region, Kazakhstan, the isolate Sheeppox/KZ/Targap/2024 was characterized using PCR and whole-genome sequencing on the Ion GeneStudio S5 platform. Phylogenetic analysis showed 99.94% nucleotide identity with isolates from the Russian Federation, demonstrating the value of genomic analysis for determining relationships among outbreak-associated strains [37]. Whole-genome data may also support vaccine matching, diagnostic assay development, and monitoring of viral evolution. In sheep, NGS can be applied to respiratory samples, skin lesions, blood, tissues, aborted fetal materials, and fecal samples, particularly when routine diagnostic assays fail to identify the causative agent. It is especially useful for detecting mixed infections and unexpected viral agents that may be missed by single-target assays. Despite these advantages, routine implementation of NGS-based surveillance remains limited by sequencing costs, workflow complexity, and the requirement for specialized analytical expertise. Therefore, its greatest current value is often observed in outbreak investigations and unexplained clinical cases rather than routine large-scale surveillance [38]. Viral metagenomics represents a valuable sequence-independent approach for novel virus discovery because it enables broad characterization of viral nucleic acids without requiring predefined target sequences. Its utility is particularly evident in unexplained outbreaks, mixed infections, and surveillance scenarios where conventional targeted assays may fail. However, metagenomic detection is influenced by viral abundance, host nucleic acid background, contamination, sequencing depth, and reference database completeness. Furthermore, the identification of viral sequences alone does not confirm active infection or disease causation; therefore, detected viral signals require validation through clinical, pathological, molecular, and epidemiological evidence [39]. Metagenomic sequencing supports virome characterization, identification of divergent viruses, reconstruction of viral genomes, and detection of co-infecting pathogens. In ovine disease investigations, metagenomics is particularly valuable when clinical signs are nonspecific or when conventional PCR and serological assays fail. For example, metagenomic sequencing has identified unexpected viruses from sheep respiratory samples, supporting its application in detecting possible spillover and cross-species transmission events in mixed farming systems [40]. However, metagenomic discovery introduces an important interpretative challenge: detection of viral sequences does not necessarily confirm active infection, pathogenicity, or disease causation. Therefore, sequence-based discoveries require validation using clinical findings, pathological evidence, viral load assessment, and epidemiological information. Nanopore sequencing is an emerging platform with increasing importance for genomic and metagenomic detection because of its portability, long-read capability, and real-time sequencing potential. These characteristics make it valuable for rapid outbreak investigation, field surveillance, and near-real-time genomic analysis in veterinary settings [41]. In ovine virus research, nanopore sequencing can support targeted sequencing, whole-genome analysis, and untargeted metagenomic detection.

Although nanopore sequencing can reduce the time between sample collection and genomic information generation, limitations related to sequencing accuracy, sample quality, host nucleic acid background, and bioinformatics requirements must be addressed before widespread veterinary application [42]. Bioinformatics analysis is a critical component of genomic and metagenomic virus detection. Sequencing data require quality assessment, host-read removal, assembly, taxonomic classification, genome annotation, and contamination evaluation. For novel ovine viruses, interpretation remains challenging because many animal viruses are underrepresented in reference databases. Therefore, candidate viral sequences should be evaluated using multiple approaches, including similarity analysis, conserved-domain identification, genome organization, and comparison with related viral groups. This represents a major bottleneck in novel virus discovery because sequencing capacity is expanding faster than biological interpretation frameworks. Improving reference databases and analytical pipelines is therefore essential for converting sequence detection into meaningful epidemiological knowledge [43]. Phylogenetic analysis provides an evolutionary framework for interpreting newly identified viruses and determining whether they represent known lineages, divergent strains, or potentially novel variants. It can support investigation of geographic origins, transmission patterns, host adaptation, recombination, and evolutionary relationships among viral strains. When combined with epidemiological information, phylogenetic analysis improves understanding of viral circulation and emergence in sheep populations [44]. However, evolutionary relationships alone cannot establish whether a detected virus is responsible for disease. Therefore, phylogenetic interpretation must be integrated with clinical, pathological, and epidemiological evidence to determine biological relevance [44]. Despite their strengths, genomic and metagenomic approaches remain limited by low viral abundance, poor sample quality, host nucleic acid background, contamination, incomplete reference databases, uneven sequencing depth, and complex analytical workflows. Furthermore, the identification of viral nucleic acids alone does not prove causality. Therefore, sequencing results should be interpreted together with clinical signs, pathology, serology, viral load measurements, and confirmatory assays such as PCR, virus isolation, or seroconversion studies [45].

Collectively, genomic and metagenomic approaches provide a framework for moving from reactive identification toward more proactive surveillance of emerging ovine viral threats. Their greatest value lies in combining virus discovery, evolutionary analysis, and epidemiological interpretation rather than replacing conventional diagnostic systems [36,39,45]. The principal strengths, limitations, and applicability of major diagnostic approaches for detecting known and novel ovine viruses are summarized in Table 2.

## 6. Surveillance and Veterinary Epidemiology

Veterinary epidemiology and integrated surveillance are essential for identifying high-risk populations, detecting emerging viral threats, tracing transmission pathways, and limiting virus spread within sheep-production systems. Viral emergence and dissemination may be promoted by animal movement, communal grazing, mixed-species farming, livestock trade, wildlife contact, and exposure to arthropod vectors. Surveillance should therefore extend beyond clinical reporting and laboratory confirmation to incorporate field observations, confirmed laboratory findings, molecular and genomic data, animal-movement records, vector surveillance, and ecological risk factors associated with suspected disease events. However, the increasing complexity of ovine viral emergence requires a transition from disease-focused surveillance toward integrated systems capable of detecting changes in pathogen circulation, host interactions, and environmental risk factors. Surveillance effectiveness depends not only on diagnostic capacity but also on timely data integration, coordinated information sharing, and the ability to translate epidemiological signals into early intervention strategies [46]. This integrated approach is particularly important for transboundary and economically significant ovine viruses, including peste des petits ruminants virus (PPRV), bluetongue virus (BTV), sheep pox virus, Rift Valley fever virus (RVFV), small ruminant lentiviruses (SRLVs), orf virus, border disease virus, and louping ill virus [47]. Passive surveillance remains the principal method of disease detection in many sheep-producing regions. It relies on farmers, veterinarians, livestock traders, slaughterhouse personnel, animal-health inspectors, and diagnostic laboratories reporting unusual clinical signs, increased morbidity or mortality, abortion outbreaks, respiratory disease, oral lesions, neurological abnormalities, skin lesions, or reduced flock productivity. Passive surveillance is relatively economical and can support early recognition of clinically apparent outbreaks of PPR, sheep pox, bluetongue, contagious ecthyma, and Rift Valley fever [48]. Nevertheless, passive surveillance is inherently reactive because it depends on disease recognition and reporting after clinical signs appear. This creates a risk of delayed detection, particularly for subclinical, persistent, or newly emerging infections. Therefore, passive surveillance should be considered an essential first layer of detection but insufficient as a standalone strategy for emerging virus preparedness [48,49]. Its effectiveness may be limited by underreporting, delayed sample submission, inadequate farmer awareness, restricted access to veterinary services, and weak compensation or outbreak-response systems. These limitations can be reduced through farmer education, standardized case definitions, mobile reporting platforms, improved access to veterinary support, and rapid sample-transport systems [49]. Active surveillance is required when the objective is to detect subclinical infection, estimate disease prevalence, identify high-risk areas, monitor vaccination, or provide evidence of freedom from disease. This approach includes serological surveys, PCR screening, abattoir sampling, market surveillance, sentinel flock monitoring, post-vaccination seromonitoring, and targeted sampling in border or outbreak-prone areas [50]. For PPRV, active serosurveillance and post-vaccination monitoring are particularly important because eradication programs require evidence of population immunity and early detection of residual virus circulation [51]. Likewise, active and repeated testing is necessary for SRLV infections because chronic infection, genetic diversity, and subclinical circulation can result in underdiagnosis when only a single diagnostic approach is applied [52]. Flock-level pestivirus monitoring is particularly relevant on farms keeping sheep or goats alongside cattle; in a Belgian study, pestivirus seropositivity in small-ruminant flocks was associated with the presence of cattle on the same farm [53]. These examples demonstrate that active surveillance provides greater sensitivity for detecting hidden virus circulation; however, it requires appropriate risk-based sampling strategies because universal monitoring of all populations is often economically and logistically unrealistic. Therefore, prioritization of high-risk populations is essential for efficient surveillance design [50,51]. Outbreak-based surveillance is triggered when clinical evidence, laboratory confirmation, or epidemiological indicators suggest active viral transmission. During outbreaks, veterinary epidemiology is essential for identifying the index case, estimating the timing and source of introduction, tracking animal movement, mapping affected holdings, and identifying populations at risk [54]. Laboratory confirmation using ELISA, antigen detection, PCR, virus isolation, or sequencing is required because many ovine viral diseases have overlapping clinical manifestations. Genomic and phylogenetic information can further distinguish local viral persistence from new introductions, particularly for PPRV, BTV, RVFV, capripoxviruses, and parapoxviruses. However, outbreak investigation provides valuable information after transmission has already occurred. Therefore, genomic epidemiology should increasingly be integrated into routine surveillance systems to support earlier recognition of viral introduction and transmission changes rather than being applied only during confirmed outbreaks [55]. Recent RVF outbreak investigations have demonstrated that rapid diagnosis, active community surveillance, slaughterhouse surveillance, vaccination, vector control, and One Health coordination can reduce outbreak impact and minimize risks of human spillover [56]. Risk-based surveillance improves resource allocation by focusing monitoring efforts on populations, seasons, and geographic regions where viral introduction or spread is more likely. Priority areas may include border regions, livestock markets, pastoral migration routes, regions with high small-ruminant density, previously affected areas, vector-rich environments, and locations adjacent to wildlife habitats [46]. Risk-based approaches can also support detection of subclinical infection in apparently healthy flocks and guide quarantine, vaccination, movement restrictions, and biosecurity interventions [51]. The major advantage of risk-based surveillance is that it transforms surveillance from uniform monitoring into a predictive approach that considers pathogen ecology, host movement, environmental conditions, and historical disease patterns [46].

Vector-borne virus monitoring is increasingly important in ovine surveillance because climate variability, rainfall patterns, irrigation, land-use change, and livestock movement can alter arthropod distribution and seasonal abundance. Bluetongue virus surveillance should include monitoring of *Culicoides* biting midges, vector density, susceptible sheep populations, circulating serotypes, and potential emergence of novel or reassortant strains [57]. RVFV surveillance should integrate livestock abortion reports, mosquito ecology, rainfall and flooding indicators, slaughterhouse testing, community reporting, and human health information [56]. Tick-borne viruses such as louping ill virus require ecological surveillance because infection risk is influenced by habitat characteristics, deer density, tick abundance, climate, and wildlife hosts [58]. The wildlife–livestock interface represents another critical component of ovine virus epidemiology. Sheep may share grazing areas, water sources, vectors, and ecological niches with wild ruminants, deer, antelope, rodents, birds, and other wildlife species. These interactions may facilitate spillover, spillback, and maintenance of viruses outside domestic sheep populations. For PPRV, wildlife infection remains an important knowledge gap for eradication planning and biodiversity protection [55]. Therefore, future ovine virus surveillance systems should incorporate domestic animals, wildlife hosts, vectors, climate variables, and environmental drivers, particularly for infections such as BTV, RVFV, and louping ill virus [58]. A major challenge for future surveillance is therefore moving from isolated pathogen monitoring toward ecosystem-based surveillance that recognizes the interconnected roles of livestock, wildlife, vectors, and environmental change in viral emergence [55,58]. Overall, surveillance and veterinary epidemiology provide the operational foundation for early warning, outbreak preparedness, and control of emerging and novel ovine viruses. The strongest systems combine passive reporting, active sampling, outbreak investigation, risk-based prioritization, vector monitoring, wildlife–livestock surveillance, and laboratory confirmation. When linked with molecular diagnostics, genomic sequencing, phylogenetic analysis, and One Health coordination, integrated surveillance becomes more than a detection system; it becomes a predictive framework capable of identifying emerging threats, tracing transmission pathways, guiding vaccination and movement-control strategies, and reducing the economic and public-health impacts of ovine viral diseases [56].

## 7. One Health Perspectives

One Health perspectives are essential for the identification, surveillance, and control of emerging and novel ovine viruses because sheep health is closely connected with human livelihoods, wildlife ecology, arthropod vectors, and environmental change. The One Health concept recognizes that the health of humans, domestic animals, wildlife, and ecosystems is interconnected and requires coordinated action across veterinary, medical, ecological, and public-health sectors. In ovine virology, this approach is particularly important because sheep may serve as susceptible hosts, indicators of viral circulation, or potential bridge hosts at livestock–wildlife–human interfaces.

**Figure 2:**
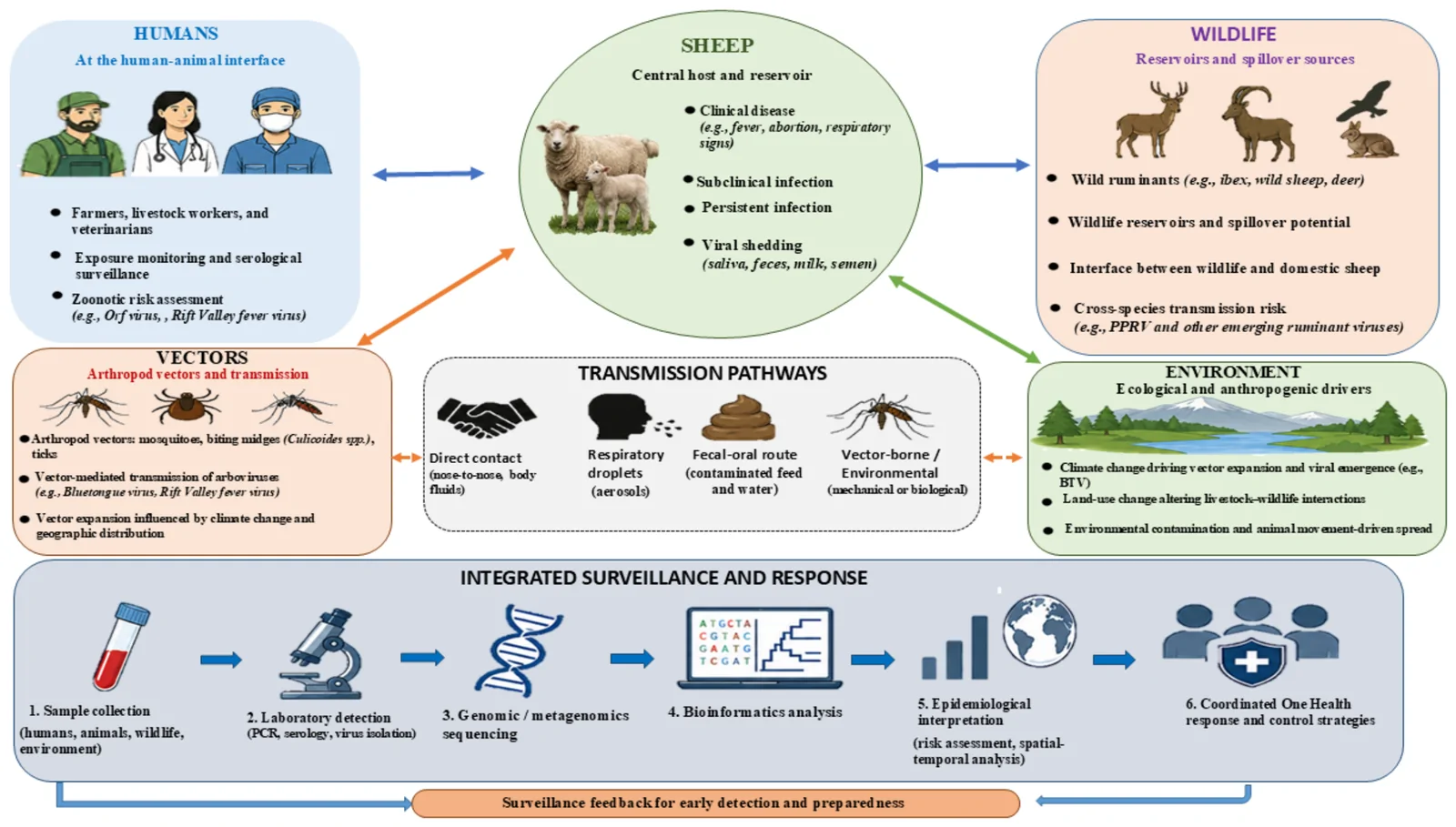
One Health framework for surveillance and response to emerging and novel ovine viruses. This framework illustrates the interactions among sheep, humans, wildlife reservoirs, arthropod vectors, environmental drivers, and transmission pathways involved in ovine virus emergence. The integrated surveillance pathway combines sample collection, laboratory diagnosis, genomic and metagenomic sequencing, bioinformatic analysis, epidemiological interpretation, and coordinated One Health responses. Arrows indicate interaction and information flow among components, colored arrows represent ecological and epidemiological connections, and the feedback loop represents the integration of surveillance findings into future detection and preparedness strategies.

However, emerging ovine viruses cannot be effectively managed through livestock-focused surveillance alone because viral emergence is influenced by complex interactions among host populations, wildlife reservoirs, vectors, environmental changes, and human activities. Therefore, One Health represents not only a collaborative framework but also an essential surveillance model for identifying and interpreting novel viral threats [59]. The interconnected relationships among sheep, humans, wildlife, arthropod vectors, environmental drivers, transmission pathways, and integrated surveillance strategies that influence emerging ovine virus detection are summarized in Figure 2.

The zoonotic importance of ovine viruses is clearly demonstrated by Rift Valley fever virus (RVFV) and orf virus. RVFV is a mosquito-borne zoonotic pathogen that causes abortion storms, neonatal mortality, and economic losses in sheep and other ruminants, while humans may become infected through mosquito exposure or contact with infected blood, tissues, aborted materials, or animal fluids [60]. These transmission pathways create occupational risks for shepherds, farmers, veterinarians, abattoir workers, laboratory personnel, and animal handlers. Recent RVFV investigations have demonstrated that early molecular detection, active community and slaughterhouse surveillance, vaccination, vector control, and One Health coordination are essential for reducing animal losses and limiting human spillover. The RVFV example demonstrates that molecular detection alone is insufficient for outbreak prevention; effective control requires integration of diagnostics with ecological monitoring, community-level surveillance, vaccination strategies, and public-health preparedness [61]. Orf virus provides another important example of the livestock–human interface. It is transmitted from infected sheep and goats to humans through direct contact with lesions, contaminated materials, shearing, lamb feeding, slaughtering, or skin injuries [62]. Although human infection is usually self-limiting, recognition among healthcare workers, veterinarians, and livestock handlers remains important because clinical presentation may resemble other poxvirus-related skin conditions. The wildlife–livestock interface represents another critical component of ovine virus emergence. Sheep frequently share grazing areas, water sources, vectors, and ecological niches with wild ruminants, deer, rodents, birds, and other wildlife species. These interactions may facilitate spillover from wildlife to domestic animals, spillback from livestock to wildlife, and maintenance of viruses within multi-host ecological systems. Wildlife infection remains an important knowledge gap for peste des petits ruminants virus (PPRV) eradication planning because susceptible wildlife species may influence virus persistence and control strategies. A major challenge for One Health surveillance is that wildlife monitoring is often less developed than livestock surveillance, limiting understanding of how wildlife reservoirs contribute to viral maintenance, emergence, and reintroduction into sheep populations [63]. Vector-borne viruses further demonstrate the need to integrate animal, human, and environmental surveillance. Bluetongue virus (BTV), although not considered a major zoonotic pathogen, is highly relevant from a One Health perspective because its epidemiology depends on interactions among sheep, wild ruminants, *Culicoides* biting midges, climate, land use, and animal movement [64]. Climate variability can influence vector distribution, seasonal activity, and the emergence or re-emergence of viral strains in sheep populations [65]. Similarly, RVFV emergence is associated with mosquito ecology, rainfall, flooding patterns, livestock density, and human exposure, emphasizing the value of climate-informed early-warning systems [61]. Tick-borne viruses such as louping ill virus also require ecological surveillance because infection risk is influenced by tick abundance, deer populations, habitat characteristics, and environmental conditions [66]. Therefore, future surveillance systems should move beyond pathogen detection alone and incorporate predictive ecological approaches that integrate climate information, vector dynamics, and host distribution to anticipate periods of increased viral risk [64,65].

One Health perspectives also require consideration of livestock production systems and value chains. Live-animal markets, transhumant movement, communal grazing, informal trade, poor biosecurity, and mixed-species farming can increase opportunities for viral dissemination and cross-species transmission. Pestiviruses, including border disease virus and bovine viral diarrhea virus, demonstrate the importance of multispecies surveillance because related viruses can circulate among sheep, goats, and cattle, causing reproductive losses, persistent infection, and productivity decline. Similarly, small ruminant lentiviruses require long-term monitoring because chronic infection, genetic diversity, and delayed clinical expression complicate detection and control. These examples highlight that viral emergence is not only a biological phenomenon but also a consequence of animal-management practices, trade networks, ecological interactions, and human behaviour. Effective One Health surveillance must therefore combine pathogen information with knowledge of production systems and transmission environments [67]. Integrated animal–human–environment surveillance is central to detecting emerging and novel ovine viruses. At the animal level, surveillance should include syndromic reporting, serology, PCR, genomic sequencing, metagenomics, and post-vaccination monitoring. At the human level, targeted awareness and monitoring are important among high-risk occupational groups, including farmers, veterinarians, abattoir workers, and laboratory personnel. Environmental surveillance should incorporate vector monitoring, climate information, wildlife assessment, and identification of high-risk ecological interfaces. The PPR Monitoring and Assessment Tool and related eradication frameworks demonstrate how surveillance, diagnostics, stakeholder engagement, and veterinary infrastructure can support progressive disease control [68]. For zoonotic and vector-borne viruses, linking veterinary surveillance with public-health systems is essential for early warning, rapid response, and effective risk communication. Overall, One Health perspectives transform ovine virus surveillance from pathogen detection alone into an integrated risk-assessment framework that connects molecular diagnostics, genomic epidemiology, ecological monitoring, and human health preparedness. Such integration is essential for identifying novel viral threats before widespread establishment, reducing zoonotic risks, and supporting sustainable control strategies in sheep-production systems [69].

## 8. Challenges and Future Directions

**Figure 3:**
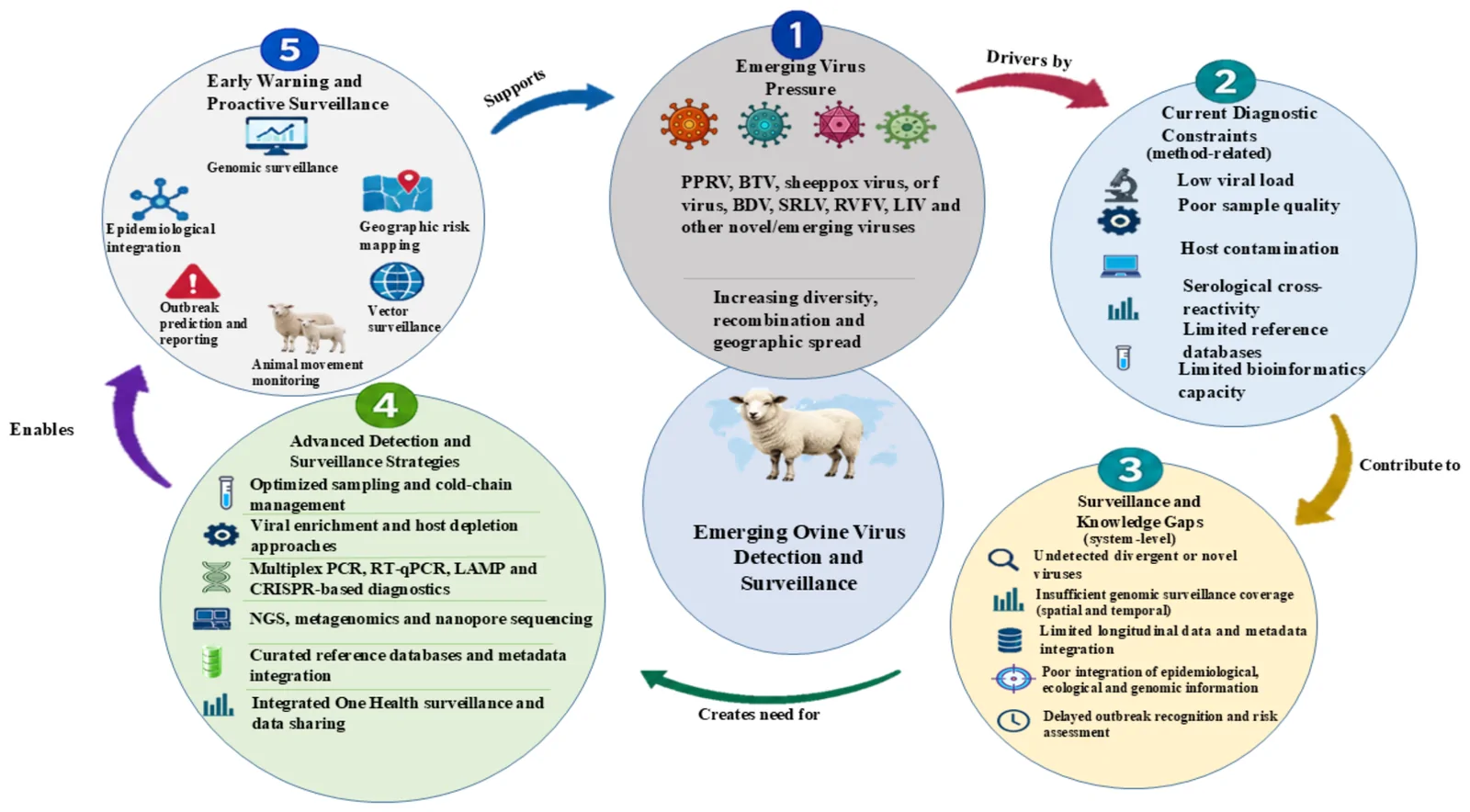
Challenges and advanced strategies for detection and surveillance of emerging ovine viruses. The framework links emerging virus pressures and diagnostic limitations with detection gaps and highlights advanced molecular technologies and integrated early-warning surveillance strategies to improve virus identification and outbreak preparedness.

Despite substantial advances in molecular diagnostics, genomic sequencing, and metagenomic surveillance, significant technical, analytical, epidemiological, and operational challenges remain in the identification of emerging and novel ovine viruses [70]. The current challenge is not only generating viral sequence information but also accurately interpreting and applying these data within biological, ecological, and epidemiological contexts for early intervention. Many ovine viral diseases share similar clinical manifestations, including fever, respiratory disease, abortion, diarrhea, oral lesions, cutaneous abnormalities, neurological signs, and reduced flock productivity. Viruses such as peste des petits ruminants virus (PPRV), bluetongue virus (BTV), sheep pox virus, orf virus, border disease virus, small ruminant lentiviruses (SRLVs), and Rift Valley fever virus (RVFV) may produce overlapping clinical syndromes, making diagnosis based solely on clinical observation unreliable [71]. Therefore, accurate detection depends on appropriate sample selection, preservation of nucleic acid quality, suitable diagnostic platforms, and sufficient laboratory capacity [70]. The major challenges and advanced strategies for improving emerging ovine virus detection and surveillance are summarized in Figure 3.

A major limitation in ovine virus detection is the difficulty of identifying infections with low viral abundance, early-stage infection, intermittent shedding, persistent infection, or partial immunity. These conditions reduce the amount of viral nucleic acid available for detection and may increase background signals from host material, bacterial sequences, and environmental contaminants. This challenge is particularly important for viral metagenomics, where low sequencing depth and high host nucleic acid background can reduce viral read recovery and generate false-negative interpretations [72]. Viral enrichment, host nucleic acid depletion, internal controls, and standardized processing workflows can improve detection sensitivity. However, these approaches may introduce taxonomic bias by preferentially enriching certain viral groups. Therefore, improving metagenomic performance requires not only laboratory optimization but also standardized sampling strategies that consider specimen type, disease stage, and epidemiological context [70,72]. Sample quality remains a practical limitation, especially in pastoral and resource-limited production systems. Variation in viral load, inhibitors, nucleic acid stability, transportation conditions, and cold-chain availability can influence diagnostic reliability. Field-adapted sampling procedures, stabilization methods, improved transport systems, and personnel training are therefore essential for increasing reproducibility and surveillance effectiveness [70]. Serological surveillance remains valuable for assessing previous exposure, estimating prevalence, and monitoring vaccination programs. However, interpretation can be complicated by delayed seroconversion, maternal antibodies, antigenic cross-reactivity, and difficulties distinguishing active infection from previous exposure. Viral genetic diversity further complicates diagnosis because assays developed against historical strains may lose performance when viral populations evolve. Genetic variation in border disease virus can influence molecular epidemiology and detection based on conserved genomic regions, while small ruminant lentiviruses show substantial genetic and antigenic diversity that may reduce diagnostic sensitivity [73,74]. These challenges demonstrate that diagnostic systems must be continuously evaluated and updated. Molecular results should therefore be interpreted together with serological data, clinical history, vaccination information, and repeated flock-level sampling when necessary [73,74,75]. Another major challenge in genomic and metagenomic surveillance is the interpretation of newly generated sequence data. Divergent or previously unrecognized viruses may remain undetected because of incomplete reference databases, geographical bias, inconsistent nomenclature, limited metadata, and underrepresentation of animal viruses. In addition, contamination and incorrect taxonomic classification may lead to inaccurate interpretation of sequencing results. Candidate viral sequences should therefore be evaluated using multiple approaches, including read-depth analysis, genome organization, conserved-domain identification, phylogenetic reconstruction, and comparison with curated databases. Future advances in virus discovery will depend not only on sequencing capacity but also on improving reference databases containing reliable information on host species, geographic origin, sample type, clinical presentation, and epidemiological context [76]. Although genomic and metagenomic approaches provide powerful tools for identifying novel viruses, detection of viral nucleic acids alone does not confirm active infection or disease causality. Viral sequences may originate from contamination, vaccine-derived material, environmental exposure, endogenous viral elements, or incidental infections. Therefore, sequencing findings should be supported by additional evidence, including PCR confirmation, viral-load estimation, serology, pathology, virus isolation, or epidemiological association where possible [71]. Emerging technologies, including portable nanopore sequencing, rapid isothermal amplification, CRISPR-based detection, biosensors, and microfluidic platforms, provide opportunities to improve field-based diagnosis. Nanopore sequencing can generate genomic information closer to outbreak locations and has demonstrated potential for rapid characterization of ovine viruses, including PPRV [77]. Similarly, RT-LAMP assays have shown promise for rapid small-ruminant disease detection without requiring conventional thermocycling equipment [78]. However, successful translation of these technologies into routine veterinary surveillance requires analytical validation, affordability, operational simplicity, quality control, and integration with existing diagnostic systems rather than technological capability alone [79,80]. Future surveillance should shift from predominantly reactive outbreak confirmation toward proactive and integrated monitoring systems. Molecular diagnostics and sequencing should be linked with serosurveillance, syndromic reporting, vector monitoring, wildlife surveillance, animal movement information, climate data, and One Health reporting networks. Priority surveillance targets include border regions, live-animal markets, pastoral migration routes, mixed-species farms, wildlife–livestock interfaces, and vector-rich environments. For RVFV, effective surveillance requires integration of livestock infection monitoring, abortion reporting, mosquito ecology, occupational exposure assessment, and environmental indicators because outbreaks affect both animal and human health [81]. Similarly, BTV surveillance requires monitoring of *Culicoides* populations, circulating serotypes, climate-associated risk factors, and potential reassortment events [82]. Ecological surveillance for louping ill virus should incorporate sheep populations, tick abundance, wildlife hosts, habitat characteristics, and land-management practices [83]. Ultimately, the future of ovine virus surveillance depends on moving from pathogen-focused detection toward predictive systems that integrate molecular diagnostics, genomic epidemiology, ecological monitoring, and One Health intelligence. Standardized sampling, affordable field diagnostics, low-cost sequencing, curated databases, and coordinated data sharing among veterinary, public-health, wildlife, and environmental sectors will be essential for early recognition and control of emerging viral threats.

## 9. Conclusions

Emerging and novel ovine viruses remain a significant challenge for animal health, livestock production, and One Health security. Although conventional diagnostics and targeted molecular assays remain essential, their dependence on known viral targets limits the detection of divergent and previously unrecognized viruses. Genomic and metagenomic approaches have expanded virus discovery and surveillance capacity; however, their effective application requires reliable sampling, improved databases, robust bioinformatics, and biological validation of detected sequences. Future ovine virus surveillance should move from reactive pathogen detection toward integrated systems combining molecular diagnostics, genomic epidemiology, veterinary surveillance, ecological monitoring, and One Health approaches. Linking viral genomic information with animal movement, wildlife interactions, vector dynamics, and environmental factors will improve early recognition, risk assessment, and preparedness against emerging viral threats in sheep.

## Figures and Tables

**Table 1:** Major Ovine Viruses and Their Relevance to Detection and Surveillance.

Virus	Importance in Sheep	Principal Detection Methods	Key Surveillance Consideration	Reference
Sheep pox virus (SPPV)	Systemic disease, skin lesions and mortality	PCR/qPCR, virus isolation, sequencing	Rapid confirmation and genomic outbreak tracing	[16]
Orf virus (ORFV)	Contagious ecthyma; zoonotic skin infection	PCR/qPCR, LAMP, serology, sequencing	Differentiation from similar skin diseases; occupational exposure	[17]
Peste des petits ruminants virus (PPRV)	Severe respiratory and gastrointestinal disease; high mortality	RT-PCR/qRT-PCR, serology, sequencing	Transboundary spread, lineage monitoring and eradication surveillance	[18]
Bluetongue virus (BTV)	Vector-borne systemic disease and production losses	RT-PCR, serotyping, sequencing	Vector expansion, serotype diversity and reassortment	[7]
Border disease virus (BDV)	Reproductive failure, congenital disease and persistent infection	RT-PCR, serology, virus-neutralization test	Persistently infected animals maintain flock transmission	[8,19]
Maedi-visna virus (MVV)	Chronic respiratory, mammary and neurological disease	ELISA, AGID, PCR/qPCR	Long incubation requires repeated flock-level testing	[9,21]
Rift Valley fever virus (RVFV)	Abortion, neonatal mortality and zoonotic infection	RT-PCR, serology, virus isolation	Integrated animal, human, vector and environmental surveillance	[10,22]
Jaagsiekte sheep retrovirus (JSRV)	Ovine pulmonary adenocarcinoma	Histopathology, immunohistochemistry, PCR	Long incubation and progressive disease hinder early detection	[11,20]
Unexpected or novel viruses	Potential emerging or cross-species viral threats	NGS, metagenomics and confirmatory assays	Sequence detection requires confirmation and biological validation	[15,24,25]

Abbreviations: AGID, agar gel immunodiffusion; ELISA, enzyme-linked immunosorbent assay; LAMP, loop-mediated isothermal amplification; NGS, next-generation sequencing; PCR, polymerase chain reaction; qPCR, quantitative polymerase chain reaction; RT-PCR, reverse-transcription polymerase chain reaction; qRT-PCR, quantitative reverse-transcription polymerase chain reaction.

**Table 2:** Comparative Performance and Operational Characteristics of Conventional, Molecular, and Sequencing-Based Methods for Ovine Virus Detection and Surveillance.

Approach	Representative Sensitivity or Performance	Turnaround and Throughput	Cost, Accessibility and Field Use	Validation, Limitations and Novel-Virus Utility	References
Virus isolation	Sensitivity depends on virus viability, specimen quality and availability of permissive cell systems	Days to weeks; low throughput	High cost; requires cell culture, specialist personnel and biosafety facilities; unsuitable for field use	Established for confirming infectious virus, but slow and insensitive when virus viability is poor; very limited unknown-virus utility without subsequent identification	[27]
Antigen detection and serology	Antigen-test performance is virus- and specimen-dependent; one PPRV study reported 75–78% nasal-swab detection and 100% specificity. Serological performance depends on infection stage, vaccination and antigenic diversity	Minutes to hours; moderate-to-high throughput	Low-to-moderate cost; widely accessible; lateral-flow tests may support field use, while ELISA supports flock screening	Validated for selected viruses; low antigen abundance, delayed seroconversion and cross-reactivity may reduce reliability; no unknown-virus utility	[28,29]
PCR, RT-PCR and qPCR	Generally high sensitivity for matched targets, but assay-, virus- and specimen-dependent	Approximately 2–6 h; moderate-to-high throughput	Moderate cost; widely available in diagnostic laboratories but usually requires extraction and thermocycling equipment	Established for validated virus-specific assays; target mismatch may cause false-negative results; low unknown-virus utility	[31]
Multiplex PCR and digital PCR	A capripoxvirus triplex qPCR reported detection limits of 5.41, 27.70 and 17.28 copies/µL for LSDV, GTPV and SPPV. Digital PCR supports low-copy detection and absolute quantification	Several hours; high throughput for multiplex panels but low-to-moderate for digital PCR	Moderate cost for multiplex PCR and high cost for digital PCR; specialized equipment required	Validation is panel- or assay-specific; multiplex competition and limited digital-PCR standardization remain concerns; low unknown-virus utility	[32,33]
LAMP and CRISPR-based assays	A PPRV RT-LAMP assay reported >97% sensitivity, 100% specificity and a detection limit of 0.3–0.8 log_10_ TCID_50_/mL. CRISPR performance is assay- and target-dependent	Commonly <1–2 h; low-to-moderate throughput	Low-to-moderate cost; potentially field-deployable using portable equipment	Validated for selected viruses, but contamination control, workflow standardization and further field validation are required; low unknown-virus utility	[34,35]
Whole-genome sequencing	Performance depends on viral load, enrichment, sample quality, genome coverage and platform	One to several days; low-to-moderate throughput	High cost; requires sequencing infrastructure and bioinformatic expertise	Established for strain characterization and outbreak tracing rather than first-line diagnosis; moderate unknown-virus utility	[37,38]
Untargeted metagenomic NGS	No universal detection limit; affected by viral abundance, host background, sequencing depth, contamination and database quality	Several days; moderate throughput	High-to-very-high cost; generally restricted to specialized laboratories	Specialized or research-oriented; independent confirmation is required; broad unknown-virus utility because no predefined target is required	[36,39,42,44]
Nanopore sequencing	Performance depends on targeted versus untargeted design, viral load, enrichment, read depth and base-calling accuracy	Hours to days; real-time analysis possible	Moderate-to-high cost; portable, but requires sample preparation, quality control and bioinformatics	Emerging and workflow-dependent; low-to-moderate unknown-virus utility when targeted and broad utility when untargeted	[40,41]

Notes: Performance values are representative and vary by virus, specimen, assay design, platform, and laboratory workflow. Cost categories reflect relative equipment, reagent, personnel, and bioinformatic requirements. Novel-virus utility was classified as Low (requires prior sequence knowledge), Moderate (detects or characterizes divergent viruses using targeted enrichment or sequencing), or High (supports sequence-independent detection of unexpected viruses). All novel findings require independent confirmation. **Abbreviations:** CRISPR, clustered regularly interspaced short palindromic repeats; LAMP, loop-mediated isothermal amplification; NGS, next-generation sequencing; PCR, polymerase chain reaction; qPCR, quantitative PCR; RT-PCR, reverse-transcription PCR; TCID50, 50% tissue culture infectious dose.

## Data Availability

No new data were created or analyzed in this study. Data sharing is not applicable to this article.

## Abbreviations

BTV	Bluetongue virus
RVFV	Rift Valley fever virus
PPRV	Peste des petits ruminants virus
SRLVs	Small ruminant lentiviruses
PCR	Polymerase chain reaction
RT-PCR	Reverse-transcription polymerase chain reaction
qPCR	Quantitative polymerase chain reaction
RT-qPCR	Reverse-transcription quantitative polymerase chain reaction
WGS	Whole-genome sequencing
NGS	Next-generation sequencing
LAMP	Loop-mediated isothermal amplification
CRISPR	Clustered regularly interspaced short palindromic repeats
ELISA	Enzyme-linked immunosorbent assay
DNA	Deoxyribonucleic acid
RNA	Ribonucleic acid
mNGS	Metagenomic next-generation sequencing
